# Efficacy of Apparent Diffusion Coefficient Sampling in Muscle Tears and Intersex Differences in Professional Football Players: A Cohort Study

**DOI:** 10.1177/19417381261492942

**Published:** 2026-10-28

**Authors:** Stefano Lusi, Giorgia Carnicelli, Alessandro Quaglia, Giulia Carimati, Piero Volpi, Marco Francone, Luca Balzarini, Nicola Magarelli

**Affiliations:** Department of Diagnostic and Interventional Radiology, IRCCS Humanitas Research Hospital, Milan, Italy, and Department of Musculoskeletal Radiology, Hôpital, AP-HP Centre, Paris, France; Department of Diagnostic and Interventional Radiology, IRCCS Humanitas Research Hospital, Milan, Italy, and Department of Biomedical Sciences, Humanitas University, Milan, Italy; Department of Knee Orthopaedic and Sports Traumatology Unit, Humanitas Research Hospital, Rozzano, Milan, Italy, and FC Internazionale Medical Staff, Milan, Italy; Department of Knee Orthopaedic and Sports Traumatology Unit, Humanitas Research Hospital, Rozzano, Milan, Italy, and FC Internazionale Medical Staff, Milan, Italy; Department of Knee Orthopaedic and Sports Traumatology Unit, Humanitas Research Hospital, Rozzano, Milan, Italy, and FC Internazionale Medical Staff, Milan, Italy; Department of Diagnostic and Interventional Radiology, IRCCS Humanitas Research Hospital, Milan, Italy, and Department of Biomedical Sciences, Humanitas University, Pieve Emanuele, Milan, Italy; Department of Diagnostic and Interventional Radiology, IRCCS Humanitas Research Hospital, Milan, Italy; Department of Diagnostic and Interventional Radiology, IRCCS Humanitas Research Hospital, Milan, Italy, and Department of Biomedical Sciences, Humanitas University, Pieve Emanuele, Milan, Italy

**Keywords:** athlete, apparent diffusion coefficient, muscle tear, sports injury

## Abstract

**Background:**

Magnetic resonance imaging (MRI) is the gold standard for monitoring the evolution of muscle injuries in athletes, although no established quantitative measure of recovery currently exists.

**Hypothesis:**

Apparent diffusion coefficient (ADC) values may provide a quantitative measure of muscle healing, and sex-related differences may exist in the evolution of muscle injuries.

**Study Design:**

Single-center retrospective observational cohort study.

**Level of Evidence:**

Level 2a.

**Methods:**

Twenty-six professional football (soccer) players (13 male ,13 female) undergoing MRI for muscle injury were retrospectively selected between October 2020 and June 2024. All athletes underwent MRI at baseline (T0) and at 2-week follow-up, including diffusion-weighted imaging (DWI) acquisitions with b values of 0-100-800 s/mm^²^. Two musculoskeletal radiologists measured ADC values within the index lesions at each examination. Changes in ADC between the two time points (ΔADC) were assessed using a paired-samples t test, while sex-related differences were evaluated using the Mann–Whitney *U* test.

**Results:**

The overall mean ADC value at the lesion site decreased from 2.06 ± 0.35 mm^2^/s at T0 to 1.61 ± 0.30 mm^2^/s at follow-up corresponding to a mean ΔADC of 0.44 ± 0.20 and a 21.84% reduction. The difference between T0 and follow-up was statistically significant (*t* = 8.12; *P* < 0.001). Mean ΔADC values were 0.32 ± 0.20 mm^²^/s in male players and 0.56 ± 0.25 mm^²^/s in female players, respectively, with a statistically significant difference between sexes (z = −2.95; *P* = 0.003). When only hamstring injuries were considered, female players showed a significantly greater decrease in ADC over time than male players (Mann–Whitney *U* test; exact *P* = 0.02), with mean ΔADC values of 0.56 ± 0.30 and 0.26 ± 0.08 mm^²^/s, respectively.

**Conclusion:**

Muscle injury is associated with an acute increase in ADC values followed by a progressive decrease during healing. ADC may therefore represent a quantitative imaging biomarker for monitoring muscle recovery.

**Clinical Relevance:**

ADC measurement within muscle tears may provide a quantitative assessment of healing status and support individualized rehabilitation strategies in athletes. Sex-related differences may exist in injury pathophysiology and recovery patterns relevant to safe return to play.

Almost one-third of all injuries in professional football (soccer) are muscle injuries, which represent a significant issue for players and their teams in terms of time lost and return to play (RTP).^1,3,4,5^ Healing is a complex process consisting of distinct phases - namely “destruction, regeneration, and remodelling” - which overall last several months.^9,23^ During this long recovery time, it is challenging to state when an athlete can safely RTP, as it depends on a variety of factors among which the severity, structures involved, and injury site.^6^ Although ultrasound is useful for the initial assessment and the dynamic diagnosis, magnetic resonance imaging (MRI) remains the method of choice for evaluating the lesion and its evolution, especially in elite settings.^10^ The standard examination involves the use of fluid-sensitive sequences such as T2-weighted imaging (T2WI), with fat saturation (FS), proton density (PD), and/or short-tau-inversion recovery (STIR) sequences to quantify edema and identify the injury site, along with T1-weighted imaging (T1WI), useful for the detection of intramuscular hematomas.^6,9,22^ Many MRI protocols have been used to assess clinical grade, but little is known about diffusion-weighted imaging (DWI) of muscle injury or whether intersex differences exist in muscle recovery and its assessment.^2,3,10,15,16^ DWI can depict cellular damage as a function of restriction of water molecule diffusivity across cells^8^; hence, its utility in visualizing skeletal muscle fiber strain and rupture.^12^ The degree of damage can be quantified in terms of apparent diffusion coefficient (ADC) - a DWI-derived parameter that expresses the degree of restriction in a certain region of interest (ROI).^8,12^ The aim of our study was to investigate the role of ADC in assessing skeletal muscle injury in professional athletes and its variation during healing. Secondly, we aimed to evaluate the impact of sex on the timing and pattern of recovery, as well as the possible resulting imaging differences. Several physiology studies highlight difference in the composition and characteristics of muscles between female and male athletes.^1,7,17^

Although the relationship between DWI and ADC and injury severity has been investigated,^16^ to our knowledge, the relationship between ADC values and sex in professional athletes has never been reported.

## Methods

This single-center observational study was conducted in accordance with the principles of the Declaration of Helsinki. Ethics approval was waived due to the retrospective observational design of the study. All patient data were anonymized; informed consent was waived due to the retrospective nature of the study.

Participants were selected retrospectively from male and female professional football players competing in Serie A and European competitions who underwent MRI for clinically suspected muscle injury between October 2020 and June 2024. Players were scanned throughout the year, regardless of the timing within the competitive season. The players’ activity included strength training, speed and acceleration exercises, endurance training, and injury prevention and/or mobility programs. Maximum strength activity involved efforts close to physical limits - typically ≥90% to 100% of their maximal capacity in terms of speed, power, and heartrate. Muscular training at maximal capacity included heavy weightlifting (1-repetition maximum [1RM]), plyometrics, short-duration high-force production exercises. All players were monitored regularly by professional trainers and a dedicated team of physicians, physical therapists, and orthopaedic specialists from Serie A teams. All imaging examinations were interpreted by the same senior musculoskeletal (MSK) radiologist with >30 years experience.

The scanned regions included the thigh - specifically the anterior compartment (the sartorius muscle, rectus, and vastus femoris muscles), the medial compartment (the gracilis, the adductor longus, adductor brevis, and adductor magnus), and the hamstring muscles (biceps femoris, semimembranosus, and semitendinosus), as well as the calf muscles (soleus, gastrocnemius, and plantaris muscles).

### Inclusion Criteria

Criteria for the selection of study participants were as follows:

- Professional football (soccer) activity.
- Age >18 years.
- Full availability of medical chart and previous imaging examinations.
- Baseline MRI acquired within ≤3 days from the occurrence of muscle injury.
- No previous history of MSK congenital or acquired pathology, or developmental variants.
- No previous injury involving the muscle or anatomical region examined.
- Availability of baseline and follow-up MRI scans compliant with the standard protocol used at our institution for the evaluation of myotendinous pathology.
- No history of knee surgery.

Because of the retrospective nature of the study, informed consent was waived. Our research was conducted in accordance with the Declaration of Helsinki.^25^

Lesion grade was assessed according to the British Athletic Muscle Injury Classification (BAMIC) score (Supplementary Table 1, available in the online version of this article).^19^ According to this system, lesions were classified into BAMIC grade 0, 1, 2, 3, or 4.

### MRI Evaluation and Scanning Protocol

Patients underwent a baseline unenhanced MRI scan (T0) within 3 days after injury and a follow-up MRI scan between the second and third week after injury, using the same protocol. This timeframe was chosen because it corresponds to the early repair phase of muscle healing, which has been described as lasting approximately 2 to 3 weeks, and allows reassessment of edema regression, connective tissue gap evolution, and early scar formation.^5,9,23^ Importantly, this follow-up interval was not intended to determine readiness for RTP, but rather to serve as part of close interim monitoring, and to provide a more comprehensive assessment of muscle recovery. MRI examinations were performed using a 1.5 T scanner (Philips Ingenia 1.5 T), located inside the hospital at ground level, inside the radiology department. The protocol included noncontrast acquisitions in the sagittal, coronal, and axial planes. Sequences obtained were a STIR, turbo-spin echo (TSE), T2WI with the Dixon method, SE, T1WI, T2WI fast-field echo (FFE), and DWI with b values of 0-100-800 and ADC maps (μm^2^/second) reconstructed using the maximum b-value data (details in Table 1).

**Table 1.** Scanning protocol and acquisition parameters

Parameter	STIR	Fat-sat T2WI	Dixon TSE T2*^a^*	Dixon TSE T1	FFE-T2WI	DWI
Duration, minutes:seconds	4:43	2:42	3:42	3:37	2:38	3:25
Mode	IR	TSE	TSE, Dixon method	TSE, Dixon method	GRE	EPI (epi factor, 75) b = 0-100-800, NSE, 5
Duration of echo, ms	50	90	90	9	18	88
Duration of repetition, ms	5550	3654	2962	464	663	3943
Slice thickness, mm	4	4	4	4	4	5
Voxel, mm	1.10 × 1.32 × 4.0	1.10 × 1.41 × 4.0	1.0 × 1.32 × 4.0	1.19 × 1.83 × 4.0	0.8 × 1.0 × 4.0	1.52 × 1.50 × 3.0
Matrix	430 × 339 × 249	320 × 521 × 49	452 × 380 × 35	252 × 246 × 250	500 × 300 × 25	450 × 450 × 150
Plane	Axial, coronal	Coronal	Axial, coronal	Axial, coronal	Sagittal	Axial

Either STIR or DIXON TSE T2WI sequences were performed based on the case. DWI, diffusion-weighted imaging; EPI, echo planar imaging; FFE, fast field echo; GRE, gradient echo; IR, inversion recovery; NSE, number of averages; SPIR, spectral presaturation with inversion recovery; STIR, short tau inversion recovery; TSE, turbo-spin echo; T2WI, T2-weighted imaging.

a Optional.

The same coil type (32-channel Philips DsTorso) was used for all examinations to ensure data homogeneity. The contralateral muscle was included in the field of view for each lesion. Patients were excluded if DWI image quality was judged suboptimal or if imaging had been performed on a scanner other than the Philips Ingenia 1.5 T.

### ADC Measurement

At each examination, the lesion was first assessed on T2-weighted fat-saturated or STIR sequences, recording the location, side, muscle involvement, and cross-sectional area. ADC values were measured by placing an ROI of 10 ± 3 mm^2^ at the level of the muscle lesion using the workstation tools for normalized ADC calculation The difference between baseline and follow-up ADC values was calculated as “Delta ADC” (ΔADC). When fluid collections were present (e.g., blood, serous content from the rupture), the ROI was placed positioned carefully to avoid those sites. Additional avoided regions included the subcutaneous tissue (for superficial lesions), tendons, and bone tissue (for deep lesions). All measurements were performed by a senior MSK radiologist with >30 years experience and a final-year radiology resident with specific training in MSK radiology.

### Statistical Analysis

Normality of data distribution was first assessed. Paired-sample *t*-tests were used to compare ADC values between T0 and follow-up, and to evaluate the association between ADC variation and the degree of muscle recovery. Kendall’s tau-b correlation analysis was performed to assess the association between injury severity (BAMIC grade) and ADC values.

The ΔADC values of male and female professional players were compared using the nonparametric Mann-Whitney *U* test. Inter-reader agreement between the 2 radiologists for ΔADC measurements was assessed using intraclass correlation coefficient (ICC).

Statistical significance was defined as *P* < 0.05 for all analyses. Statistical analysis was performed using Stata/SE Version 18.0 (StataCorp 2023, Stata Statistical Software, Release 18).

## Results

A total of 26 players (male, n = 13, mean age, 27.07 ± 1.5 years; female, n = 13, mean age, 28.076 ± 1 years) met the selection criteria for the study and were included between October 2020 and June 2024. In regard to ethnicity, 84.7% players were classified as White, 7.7% were Black, 3.8% were North-African, and 3.8% Latin American. Study participants were excluded (n = 104) when the MRI protocol was not compliant with the standards set in the study plan, when DWI quality was poor or when images when acquired on a different scanner than the one selected for the study; also when participants were <18 years old, in case of previous injury in the examined region or surgery, or when scanning had occurred out of the time window established in the protocol.

MRI was performed immediately after (T0, from 24 to 76 hours) and at a mean of 17.7 ± 7.5 days from initial injury (T1, follow-up), to assess muscle recovery in grade 1 lesions. In grade 2 and 3 lesions, a second follow-up MRI was performed at 30 days from the first scan to monitor the evolution of muscle injury.

BAMIC grade 1 lesion was reported in 22 athletes (11 male and 11 female); BAMIC grade 2 occurred in 2 participants (1 male and 1 female); BAMIC grade 3 lesion was reported in 1 male and 1 female professional player (Figures 1-3). Affected muscles were the biceps femoris (n = 3), the gastrocnemius (n = 2), the rectus femoris (n = 6), semimembranosus (n = 7), semitendinosus (n = 2) muscles, and the soleus muscle (n = 6). The distribution of injury sites by sex is shown in Supplementary Table 2 (available online); Fisher’s exact test reported no association between affected muscle and severity of the lesion (BAMIC) (*P* = 0.6).

**Figure 1.**
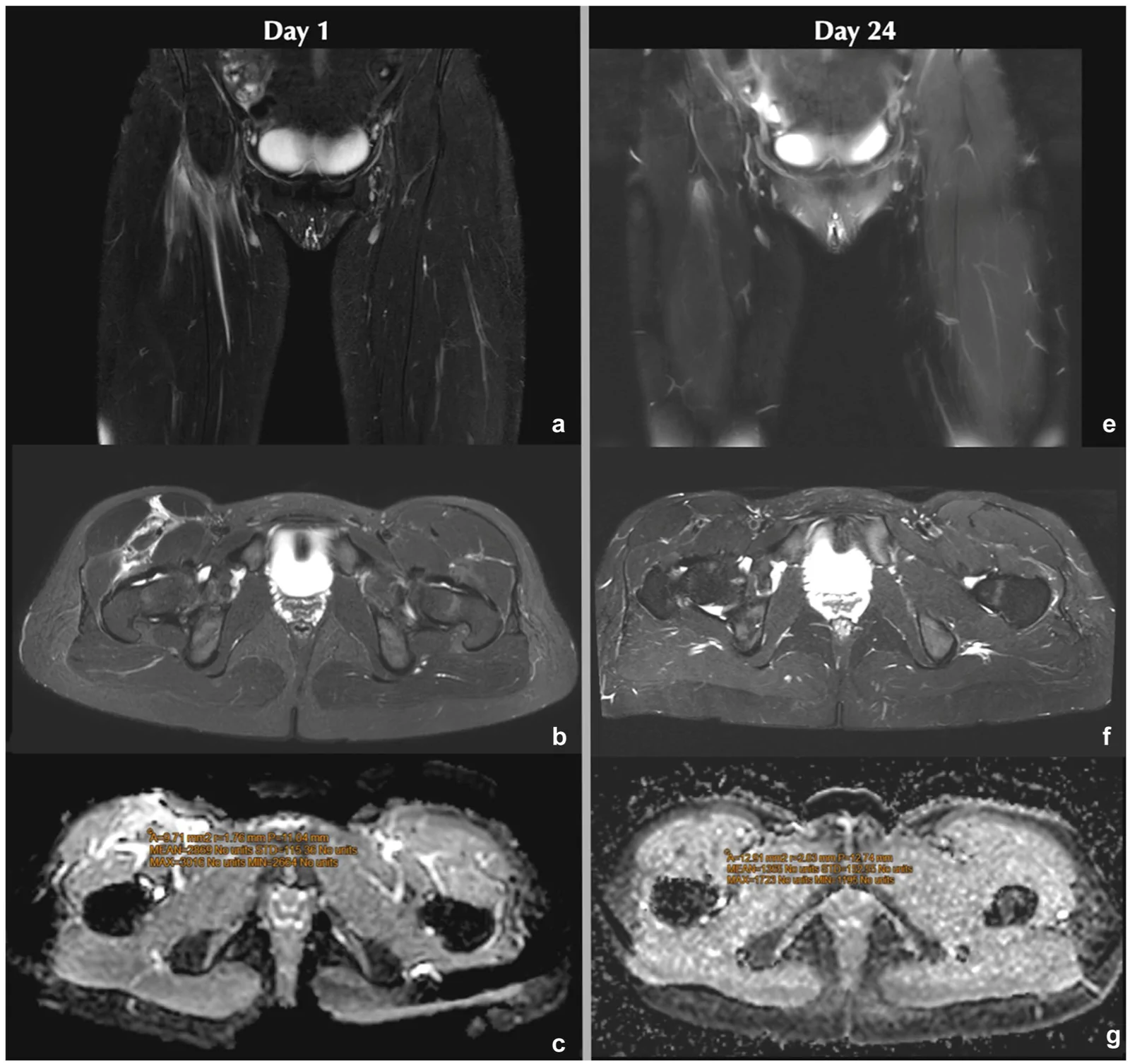
Grade 2b injury in a 26-year old female football player. MRI scans were acquired at (a-c) day 1 from injury (left side) and at (e-g) day 24 (follow-up, right side). (a, b, e, f) Axial and coronal fat-saturated TSE T2WI depict a partial-thickness myotendinous lesion at the level of the right proximal rectus femoris muscle. (c, g) Values of ADC were measured at the same level at T0 (ADC value_b800_ = 2.87 mm^2^/second; c) and at follow-up (ADC value_b800_ = 1,365 mm^2^/second; g). ADC, apparent diffusion coefficient; MRI, magnetic resonance imaging; TSE, turbo spin echo; T0, baseline; T2WI, T2-weighted imaging.

**Figure 2.**
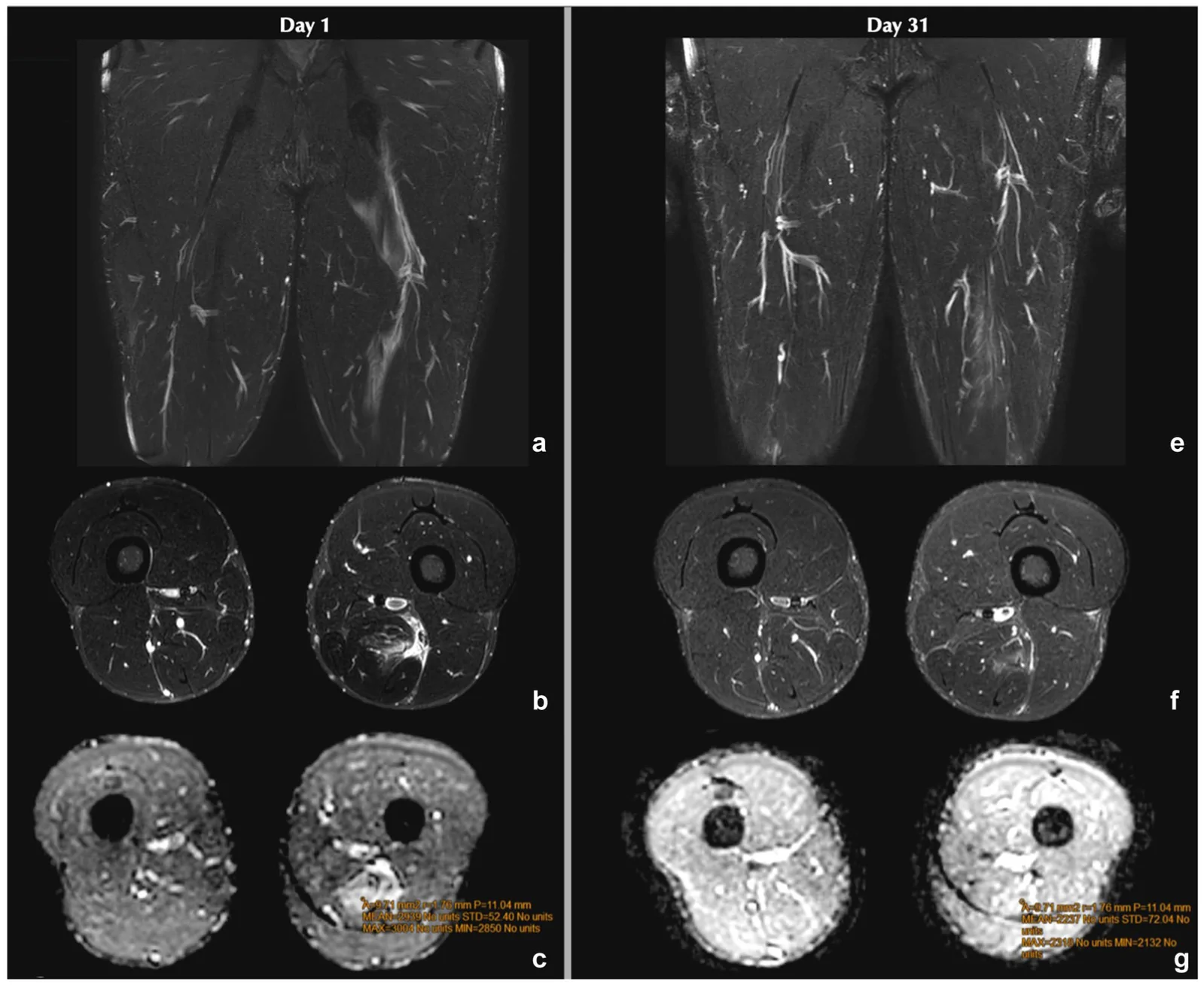
Grade 2 hamstring injury in a 27-year old male football player. MRI scans were acquired at (a-c) day 1 from injury (left side) and at (e-g) day 31 (follow-up, right side). (a, b, e, f) Axial and coronal fat-saturated T2WI depict a muscle tear at the level of the mid-distal semimembranosus muscle, where feather-shaped high signal intensity and waviness of the involved tendon can be seen. (c, g) DWI acquired at days 1 and 31 shows increased signal intensity at the lesion site. Values of ADC were measured at the same level at T0 (ADC value_b800_ = 2.44 mm/s^2^; c) and at 31 days of follow-up (ADC value_b800_ = 1.89 mm/s^2^; g). ADC, apparent diffusion coefficient; DWI, diffusion-weighted imaging; MRI, magnetic resonance; T0, baseline; T2WI, T2-weighted imaging.

**Figure 3.**
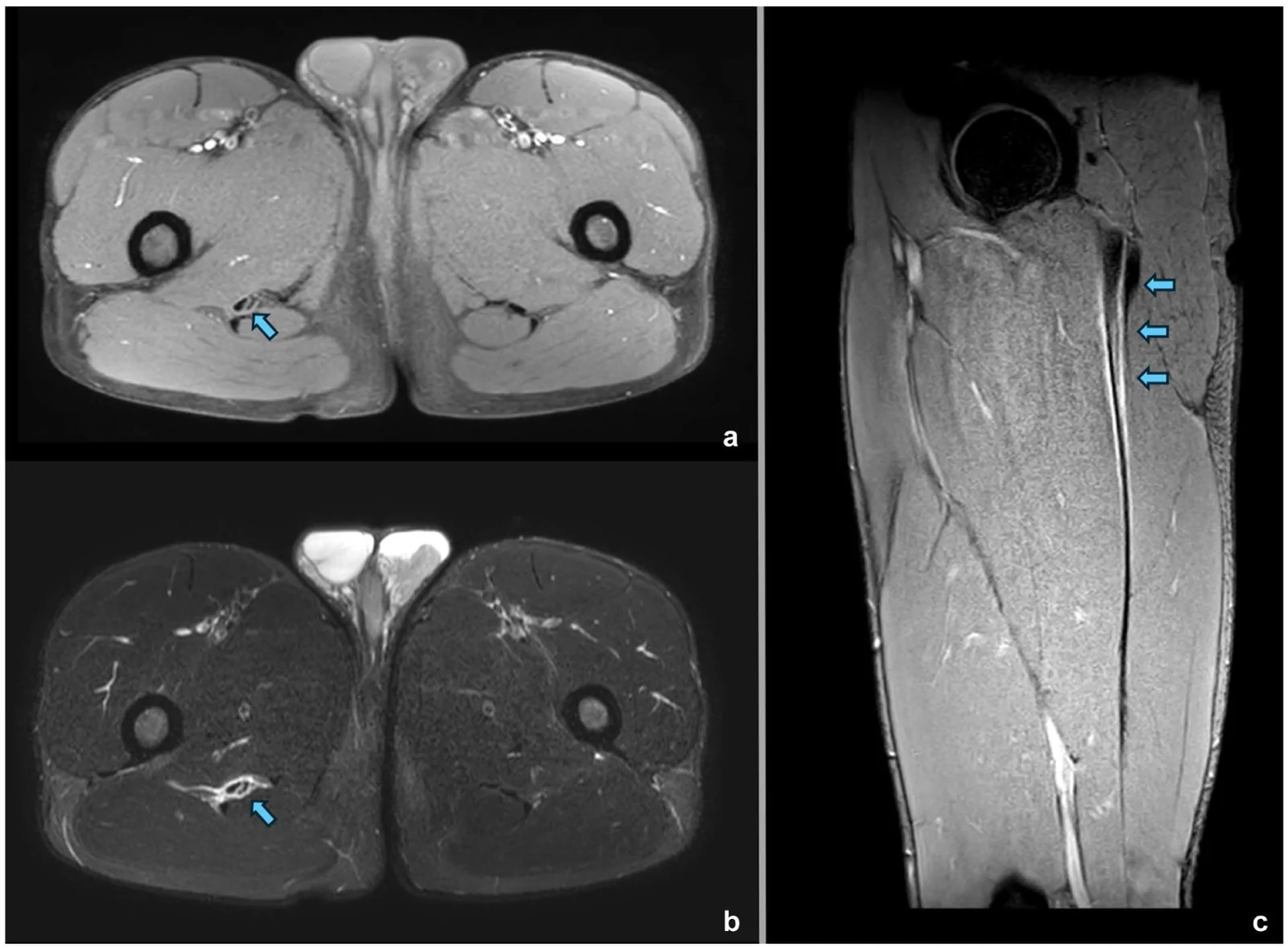
Grade 2c hamstring lesion in a 19-year-old male football player at T0. (a, b) Axial STIR and GRE-T1 fat-saturated images depict a pure tendinous lesion with partial tearing of the tendon fibers (arrows). (c) The sagittal GRE-T1 fat-saturated image depicts the lesion extending along the proximal third of the semimembranosus muscle and splitting the tendon into 2 distinct components (arrows). ADC value at T0 at the lesion site was 1.7 mm/s^2^. ADC, apparent diffusion coefficient; FS, fat saturation; GRE, gradient echo; STIR, short-tau-inversion recovery; T0, baseline.

In the overall population sample, the mean ADC value at the lesion site at baseline examination (T0) was 2.06 ± 0.35 mm/s^2^, and at follow-up it was 1.61 ± 0.30 mm/s^2^ with a mean ΔADC in the study group of 0.44 mm/s^2^ ± 0.2 (n = 26), being equal to a 21.84% reduction compared with baseline; the difference between ADC at T0 and at follow-up is depicted in Figure 4 (boxplot). According to the paired-sample *t*-test, the difference in the ADC value between T0 and follow-up was significant, with *t* = 8.12, *P* < 0.001, and effect size expressed as Cohen’s *d* = 1.35. Kendall’s tau-b correlation analysis showed no statistically significant association between the severity of muscle injury (BAMIC grade) and the ADC value measured at the injured site at T0 (τb = 0.211; *P* = 0.22)

**Figure 4.**
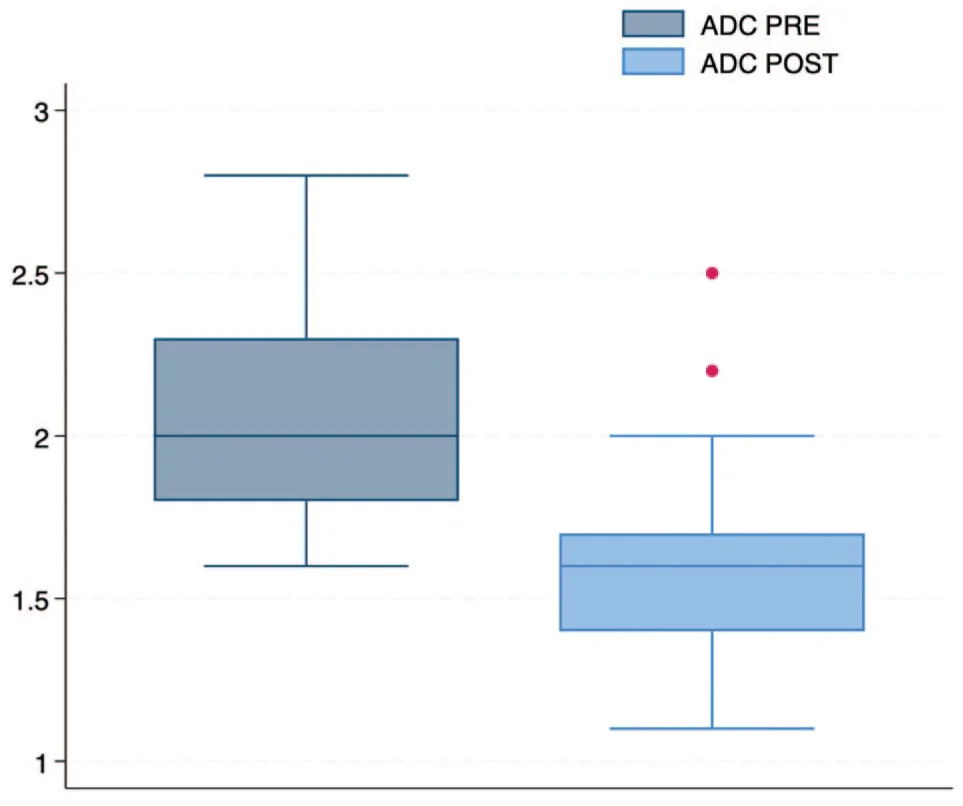
Boxplots depict the mean values of ADC (mm^2^/second) and relative standard deviation at T0 (ADC PRE) and at follow-up (ADC POST) in the overall population sample; a significant difference was found between the initial assessment and the follow-up. ADC, apparent diffusion coefficient; T0, baseline.

In male professional players, the mean ADC value measured at the lesion site by the more experienced radiologist at T0 evaluation was 1.94 ± 0.26 mm^2^/second, and at first follow-up it was 1.61 ± 0.81 mm^2^/second, corresponding to a 17.01% decrease and a mean ΔADC value of 0.32 ± 0.2 mm^2^/second. In female professional players, the mean ADC value at T0 evaluation was 2.2 ± 0.38 mm^2^/second and at first follow-up it was 1.60 ± 0.34 mm^2^/second, corresponding to a 27.28% decrease and a mean ΔADC of 0.56 ± 0.25 mm^2^/second.

The female and male subgroups were comparable in terms of population characteristics, pattern, and site of injury; however, the distribution of ΔADC values was non-normal; hence, nonparametric testing was chosen for assessing intergroup differences (Supplementary Figure 1, available online). The difference in the mean ΔADC between the 2 subgroups was statistically significant according to the Mann-Whitney *U* test, *z* = −2.95, *P* = 0.003, *d* = 0.92. Figure 5 depicts the variation of each group at T0 and follow-up. Reinjury did not occur in any of the professional athletes in the study population.

**Figure 5.**
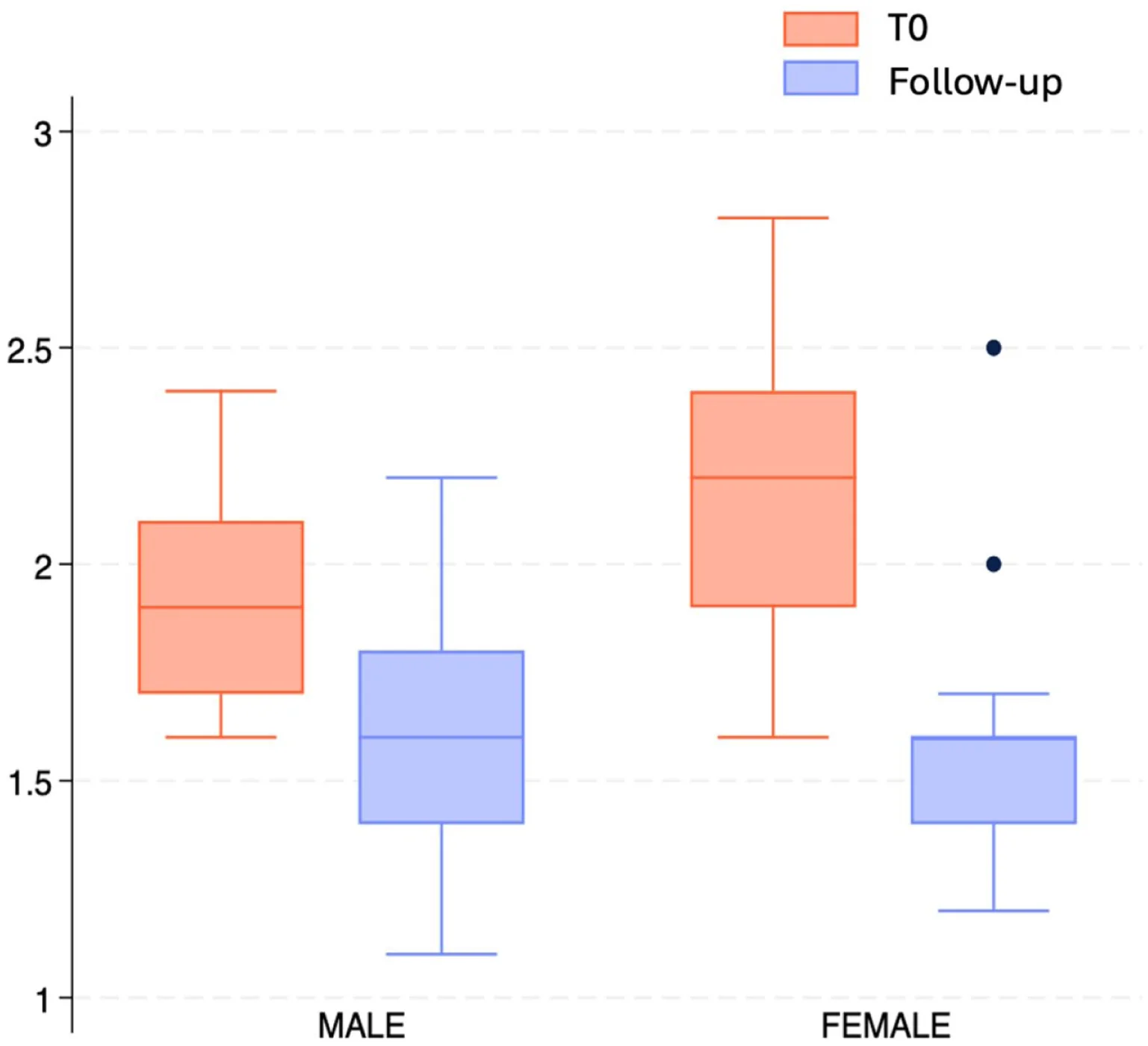
Boxplots display mean values (mm^2^/second) of ADC at T0 and at follow-up in the male and female groups. ΔADC between the 2 subgroups was statistically significant (Mann-Whitney *U* test, *z* = −2.95, *P* = 0.003). ADC, apparent diffusion coefficient; T0, baseline.

The second experienced reader (final-year resident with MSK training) reported an overall mean ADC value at the lesion site at baseline examination (T0) of 1.99 ± 0.29 mm^2^/second, and of 1.61 ± 0.27 μm^2^/second at follow-up, with a statistically significant difference between T0 and recovery (paired-sample *t* = 9.68, *P* < 0.001, *d* = 1.61). According to the less experienced reader, in male professional players the mean ΔADC value was 0.32 ± 0.2 mm/s^2^; in female professional players the mean ΔADC value was 0.49 ± 0.21 mm^2^/second, and the 2 groups had a statistically significant difference with *z* = −2.324, *P* = 0.02, *d* = 0.65 according to the Mann-Whitney *U* test. Inter-reader agreement on ΔADC values measured by the experienced radiologist and the final-year radiology resident was excellent, with an ICC of 0.89 for ADC delta measurements; detailed measurements from the second reader are reported in Supplementary Table 2 (available online).

When only hamstring injuries were considered, excluding all the other muscles of the lower limb where BAMIC score applies less reliably (12 participants with hamstring lesions: 6 male and 6 female players), findings were repeatable. In the overall population sample (n = 12), the mean ADC value at the lesion site at baseline examination (T0) was 2.08 ± 0.28 mm^2^/second, and at follow-up (T1) it was 1.66 ± 0.26 mm^2^/second with a mean ΔADC in the study group of 0.41 ± 0.07 mm/s^2^ (n = 12); the variation between T0 and T1 was statistically significant according to the paired-sample *t* test (*t* = 5.43; *P* < 0.002, *d* = 1.57).

According to intersex analysis, female players (n = 6) showed a significantly higher decrease in ADC value over the follow-up time (ΔADC), compared with male athletes (n = 6) (Mann-Whitney *U*, exact *P* = 0.02). In particular, the mean ΔADC in female players was 0.56 ± 0.30 mm/s^2^, while in male players the mean ΔADC was 0.26 ± 0.08 mm/s^2^.

## Discussion

We report DWI changes observed in 26 professional male and female athletes, each pair having the same injury pattern, severity, and site. We observed that muscle injury produced a consistent increase in ADC values at the injured site, with an average value of 2.06 mm/s^2^ in the overall cohort. This finding is coherent with previous studies on inflammatory muscle diseases and myopathies, where fiber damage is associated with increased ADC values and diffusivity at the lesion site, likely proportional to the extent of fiber disruption.^19-21^ The increase in ADC values may be related to the destruction of cellular barriers in muscle fibers, which normally restrict water movement, as also demonstrated in histopathologic studies.^13,14,24^ In addition, a hallmark of muscle injury or strain is the presence of edema, which may further explain the increased water diffusivity observed in our sample.^1,23^

We also observed a statistically significant variation in ADC values between the time of injury and follow-up, corresponding to an approximate 21% decrease from baseline in the overall cohort. We posit that the relationship between muscle healing and ADC reduction may not be direct; rather, ADC changes may reflect reductions in edema and extracellular fluid, hemorrhage resorption, and changes in tissue perfusion that accompany the healing process.^1^ When interpreted within these limitations, ADC may serve as an indirect biomarker of muscle injury in professional athletes and as a quantitative indicator of recovery. Currently, the strategies used to estimate lesion severity and prognosis rely largely on the subjective assessment of radiologists, based on the reduction of hyperintensity on T2-weighted or STIR images.^9,11^ These approaches may lack reliability and do not provide a quantitative correlation with injury.^20^ The introduction of serial ADC measurements during follow-up could hence provide a quantitative, indirect estimate of muscle healing, potentially helping clinicians to better estimate recovery timing. To our knowledge only a limited number of studies have investigated the relationship between DWI-derived parameters and muscle tears.^2,15,16^

Inter-reader agreement for ADC measurements was excellent, with significant consistency between observers. This finding suggests that ADC sampling may remain relatively stable across readers with different levels of experience.

Sex-based analysis in 2 matched cohorts showed that female athletes in our cohort had a greater variation in ADC values between injury and follow-up. Specifically, female athletes demonstrated an average ADC decrease of 27.28% at 2 to 3 weeks, compared with 17.01% in male athletes, together with slightly higher baseline ADC values at T0. Although this finding may suggest sex-related differences in the pathophysiology of muscle injury, the available data are insufficient to support this hypothesis. ADC measurements are subject to substantial variability and may be influenced by anatomical and sampling-related factors, including the number of muscle fibers arranged in series in a given muscle and the absolute length of the muscle assessed.^18,21^ These factors may differ between male and female athletes without necessarily implying a true pathophysiological difference in muscle injury.

Further research is required to properly test the hypothesis that the difference observed in ΔADC between male and female athletes resides in inherent differences in injury response. Several physiological studies have already highlighted sex differences in muscle physiology and injury response.^1,7,17,26^ Notably, women tend to have a higher proportion of type I slow-twitch muscle fibers and demonstrate greater resistance to fatigue during prolonged contractions, whereas men generally have a higher proportion of type II fast-twitch anerobic fibers.^1,7,17^ An experimental mouse model study from You et al^26^ also investigated sex differences in skeletal muscle regeneration after injury. Their findings demonstrate that the female skeletal muscle required significantly shorter regeneration time compared with male muscle, likely due to differential myosin isoform expression. Female athletes also showed faster restoration of strength and contractility compared with male athletes, with more robust recovery of whole muscle mass, twitch muscle force, and tetanic muscle force.^26^ In line with these observations, our preliminary results may suggest intrinsic sex-related differences in the pattern and timing of muscle healing, although this hypothesis requires confirmation in future studies.

## Limitations

The main limitations of our study are its retrospective design and the relatively small sample size, which represents a highly selected elite subgroup of the athletic population. In addition, the spatial resolution of ADC maps is inherently lower than that of conventional anatomical MRI sequences, and ADC measurements may not be sufficiently reproducible or fully representative of the status of the whole muscle. This may have introduced measurement inaccuracies, particularly because physiological studies have shown that muscles are not spatially uniform and that healing may vary along the length of the lesion.^21^ Furthermore, the strict inclusion criteria used to match lesions between participants, together with the strict requirements for DWI quality, may have introduced selection bias. Because athletes were scanned only after injury, baseline ADC values of the affected muscles were not available for comparison. We also did not assess whether these findings are applicable to muscles in other anatomical regions. As all participants were elite professional soccer players, the generalizability of our findings to the broader athletic population may therefore be limited.

## Conclusion

The findings of this study suggest that muscle tears in professional athletes are associated with significant variations in ADC values at the lesion site during follow-up. ADC may therefore be considered in future research as a quantitative measure to support objective monitoring of recovery. The availability of a quantitative parameter associated with muscle recovery could be useful for personalizing rehabilitation, reducing the risk of reinjury, and supporting more objective RTP decisions. Importantly, such an approach may also have relevant implications for medicolegal issues, time lost from competition, and the justification of recovery time, potentially reducing external pressure for rapid RTP (Strength of Recommendation Taxonomy, SORT Level 2 evidence).

As a collateral finding, we observed that female athletes showed a more rapid reduction in ADC values during recovery than male athletes, with significantly higher ΔADC. Future studies with focused methodology are required to determine whether inherent biological differences between male and female athletes contribute to these findings. Sex-related differences should be taken into account in elite sports to personalize athlete care.

Our findings lay the groundwork for a deeper understanding of the role of imaging biomarkers in the assessment of muscle injury. In the future, imaging-based assessment of reinjury patterns may also help identify reliable quantitative thresholds for determining safe RTP.

## Supplemental Material

sj-docx-1-sph-10.1177_19417381261492942 – Supplemental material for Efficacy of Apparent Diffusion Coefficient Sampling in Muscle Tears and Intersex Differences in Professional Football Players: A Cohort Study Supplemental material, sj-docx-1-sph-10.1177_19417381261492942 for Efficacy of Apparent Diffusion Coefficient Sampling in Muscle Tears and Intersex Differences in Professional Football Players: A Cohort Study by Stefano Lusi, Giorgia Carnicelli, Alessandro Quaglia, Giulia Carimati, Piero Volpi, Marco Francone, Luca Balzarini and Nicola Magarelli in Sports Health
