# Supplementary material for: Efficacy of Apparent Diffusion Coefficient Sampling in Muscle Tears and Intersex Differences in Professional Football Players: A Cohort Study
Source: Sports Health. 2026 Oct 28:19417381261492942. Online ahead of print. doi: 10.1177/19417381261492942 (PMC13719679; doi:10.1177/19417381261492942)
Supplement: Supplementary file 1 [file sj-docx-1-sph-10.1177_19417381261492942.docx]

**Efficacy of Apparent Diffusion Coefficient Sampling in Muscle Tears and Intersex Differences in Professional Football Players: A Cohort Study**

**Supplementary Materials**

**Supplementary Table 1.** *schematic illustration of the “British Athletics Muscle Injury Classification” (BAMIC) from Pollock et al. (2014). MRI= magnetic resonance imaging; DOMS= delayed-onset muscle soreness; STIR= short-tau inversion recovery*

# **Supplementary Table 2***. Data collected for each player: age (years), muscle affected, side, BAMIC grade, interval time between scans (days), ADC values measured by reader 1 and reader 2 at each timepoint (ADC values R1, R2) and relative* *Δ ADC. Values of ADC in male and female soccer players are expressed as μm2/s. R1= Reader 1 (i.e. experienced musculoskeletal radiologist); R2= Reader 2 (final-year resident with a training in musculoskeletal radiology); ST= semitendinosus; SM= semimembranosus; BF= biceps femoris; RF= rectus femoris; S= soleus; M GM= medial gastrocnemius; L GM= lateral gastrocnemius; p= proximal; mp= mid-proximal; md= mid-distal; d= distal; R= right; L= left;ADC= apparent diffusion coefficient*

| **Athlete** | **Age** | **Muscle** | **Side** | **BAMIC grade** | **Interval between scans** | **ADC values R1** | **Δ ADC R1** | **ADC values R2** | **Δ ADC R2** |  |
| --- | --- | --- | --- | --- | --- | --- | --- | --- | --- | --- |
| **Male 1** | 29 | S, mp | L | Ia | 23 | 1.7, 1.4 | 0.3 | 1.7, 1.4 | 0.3 |  |
| **Female 1** | 31 | S, mp | L | Ia | 21 | 2.3, 1.5 | 0.8 | 2.3, 1.6 | 0.7 |  |
| **Male 2** | 32 | S, mp | R | Ib | 13 | 1.8, 1.1 | 0.7 | 1.7, 1.2 | 0.5 |  |
| **Female 2** | 33 | S, mp | R | Ib | 16 | 2.8, 2.5 | 0.3 | 2.7, 2.5 | 0.2 |  |
| **Male 3** | 31 | ST, mp | R | Ia | 14 | 1.9, 1.6 | 0.3 | 1.8, 1.6 | 0.2 |  |
| **Female 3** | 28 | ST, mp | R | Ia | 16 | 2.4, 1.4 | 1.0 | 2.1, 1.5 | 0.6 |  |
| **Male 4** | 23 | BF, m | R | Ia | 27 | 1.7, 1.5 | 0.2 | 1.7, 1.5 | 0.2 |  |
| **Female 4** | 25 | BF, m | R | Ia | 24 | 2.0, 1.6 | 0.4 | 2.1, 1.7 | 0.4 |  |
| **Male 5** | 20 | RF, p | R | IIc | 28 | 1.9, 1.8 | 0.1 | 1.9, 1.7 | 0.2 |  |
| **Female 5** | 24 | RF, p | R | IIc | 28 | 2.8, 1.6 | 0.8 | 2.7, 1.7 | 0.9 |  |
| **Male 6** | 29 | SM, m | R | Ia | 16 | 2.4, 2.2 | 0.2 | 2.2, 2.0 | 0.2 |  |
| **Female 6** | 26 | SM, m | R | Ia | 19 | 2.3, 2.0 | 0.3 | 2.1, 1.9 | 0.2 |  |
| **Male 7** | 19 | SM, mp | R | Ia | 20 | 1.7, 1.3 | 0.4 | 1.7, 1.4 | 0.3 |  |
| **Female 7** | 21 | SM, mp | R | Ia | 18 | 1.9, 1.4 | 0.5 | 1.8, 1.5 | 0.3 |  |
| **Male 8** | 29 | M GM, d | L | Ia | 14 | 2.4, 1.6 | 0.8 | 2.2, 1.7 | 0.5 |  |
| **Female 8** | 30 | M GM, d | L | Ia | 15 | 2.2, 1.4 | 0.8 | 2.1, 1.5 | 0.6 |  |
| **Male 9** | 30 | RF, p | L | Ia | 12 | 1.9, 1.7 | 0.2 | 1.9, 1.6 | 0.3 |  |
| **Female 9** | 26 | RF, p | L | Ia | 12 | 1.7, 1.3 | 0.4 | 1.7, 1.2 | 0.5 |  |
| **Male 10** | 27 | SM, md | L | Ia | 31 | 2.1, 1.8 | 0.3 | 2.0, 1.8 | 0.2 |  |
| **Female10** | 31 | SM, md | L | Ia | 15 | 2.6, 1.7 | 0.9 | 2.5, 1.7 | 0.8 |  |
| **Male 11** | 26 | S, m | L | Ia | 12 | 2.0, 1.7 | 0.3 | 1.9, 1.6 | 0.3 |  |
| **Female11** | 30 | S, m | L | Ia | 14 | 2.1, 1.6 | 0.5 | 2.0, 1.7 | 0.3 |  |
| **Male 12** | 27 | RF, p | R | IIIc | 18 | 1.6, 1.4 | 0.2 | 1.7, 1.4 | 0.3 |  |
| **Female12** | 26 | RF, p | R | IIIc | 23 | 1.6, 1.2 | 0.4 | 1.6, 1.2 | 0.4 |  |
| **Male 13** | 34 | BF, m | R | Ia | 18 | 2.1, 1.9 | 0.2 | 1.9, 1.8 | 0.1 |  |
| **Female13** | 31 | BF, m | R | Ia | 20 | 1.9, 1.6 | 0.3 | 1.9, 1.5 | 0.4 |  |

**Supplementary Figure 1.** *Non-parametric distribution of the ΔADC values(μm^2^/s) in the general population, displayed in a histogram plot.*
